# Access to Support Services for Newcomer Youth Through the Process of School Integration: A Critical Narrative Literature Review

**DOI:** 10.1177/08295735221130442

**Published:** 2022-10-14

**Authors:** Linnea F. Kalchos, Anusha Kassan, Laurie Ford

**Affiliations:** 1 The University of British Columbia, Vancouver, BC Canada

**Keywords:** school integration, critical narrative review, critical social justice lens, qualitative research methods, culture/cross-cultural

## Abstract

While the numbers of newcomer youth continue to grow in Canada, few studies have sought to critically capture experiences of school integration and access to school-based psychosocial support services during their transition. Guided by a critical social justice lens, this research seeks to investigate issues of inequity due to the marginalization of newcomer youth in schools. The intent of this critical narrative review is to summarize, interpret, critique, and synthesize what is currently known about the ways newcomer youth access and experience school-based psychosocial support services (S-BPSS) throughout the process of school integration. This paper presents the critical narrative literature review process, a discussion of the themes that emerged from the review, and a discussion of the literature within the context of school integration. The following themes that underscore the experiences of newcomer youth accessing school-based psychosocial support services were identified: (a) underutilization/discrimination, (b) culture shift, (c) transition planning, and (d) lived experience. Important directions for future research, including the importance of studies that prioritize the perspectives of newcomer youth themselves and the implications of their lived experience for S-BPSS are provided.

In 2019, permanent and non-permanent immigration represented over 80% of Canada’s population growth (Immigrants, Refugees, and Citizenship Canada, 2020a). Moreover, in 2021 the Canadian Government was expected to admit over 405,000 newcomers to Canada, exceeding its target for the year (Immigrants, Refugees, and Citizenship Canada, 2020). As these numbers indicate, Canada is a nation built upon a foundation of immigration. Not only are many newcomers coming to Canada during a worldwide downturn of movement across borders, but the government is continuing to seek immigrants for Canada and implement programming for refugees and asylum seekers. Encouraging newcomers to come to Canada is a key piece of immigration policy, but beyond developing our workforce, it represents a growing nation of newcomers with varied backgrounds, languages, identities, and parallel needs. As immigration is expected to continue to grow with the reopening of borders and international travel, the number of newcomer youth will also continue to increase as a subset of newcomer arrivals in Canada. Children and youth with an immigrant background are expected to represent 39–49% of the total population by 2036 (Statistics Canada, 2017a).

Newcomer youth are defined as individuals who have migrated to a new country within the last 5 years, including immigrants, refugees, and international students aged 15–24 (Immigrants, Refugees, and Citizenship Canada, 2018; United Nations Department of Economic and Social Affairs [UNDESA], 2008) . This group represents an important demographic for building Canada’s future, but they face distinct challenges as a minoritized population. Schools are significant transition settings for newcomers; therefore, support is needed to facilitate school integration and provide support for newcomer children and youth (Gallucci & Kassan, 2019; Stewart, 2014). The adjustment of newcomer youth to the school system is not new to Canada; however, this phenomenon has changed over time based on various understandings. Early conceptions of school integration conceptualized newcomer youths’ adjustments as being reflected in academic self-concept and academic motivation (Areepattamannil & Freeman, 2008). School integration was also previously conceptualized as a process of acculturation whereby newcomer youth adjust and assimilate to the cultural, social, and academic norms of the new society in which they find themselves (Wang & Mallinckrodt, 2006).

Presently, the concept of school integration is referred to as the academic, social, emotional, relational, familial, and communal adjustment of newcomer youth both inside and outside of the school setting (Gallucci & Kassan, 2019). School-based psychosocial support services (S-BPSS), defined as additional support programs or services utilized outside of the classroom to support and aid the process of school integration in schools, can be key to facilitating this adjustment and transition for newcomer youth. This support can include assistance from teachers, and psychosocial support services, including counselors, English as an additional language programs and social groups, which operate inside or outside of schools to support the transition of newcomer youth. S-BPSS can also be expanded to include settlement resources, health services, summer and after-school programs, recreational activities, service clubs, youth agencies, and mentoring programs if they are offered through schools (Rossiter & Rossiter, 2009).

As newcomer communities in Canada continue to rise, a growing body of research investigating their subjective experiences has also developed in recent years (Crooks et al., 2021; Gallucci & Kassan, 2019; Kassan et al., 2019; Matejko et al., 2021; Selimos et al., 2018). We conducted a critical narrative literature review of the extant research on newcomer youth going through school integration and accessing S-BPSS. Accessing appropriate services is a crucial component of successful school integration for newcomer youth. Current research has revealed multiple barriers being faced by newcomer youth seeking to access services, and their needs are both varied and complex (Ángeles, 2021; J. Li, 2010; Oikonomidoy, 2015; Patel et al., 2016; A. C. Smith et al., 2022; Thomson et al., 2015). Psychosocial support services in schools are consistently underutilized by newcomer youth (Thomson et al., 2015). Understanding the barriers that newcomer youth face as they engage with school-based psychosocial support services during school integration provides an inventory of these challenges and highlights next steps in future research.

Regardless of the current barriers facing newcomer youth in accessing support services, several studies affirmed the importance of supportive school environments that included culturally responsive school-based psychosocial services to positively assist them in their adjustment (Bajaj & Suresh, 2018; Deckers & Zinga, 2010; Rousseau et al., 2005; Selimos & Daniel, 2017). When barriers to access are addressed and school-based psychosocial supports are effective, schools can become a place of support for newcomer youth (Bajaj & Suresh, 2018). To improve support services, researchers, school staff, and clinicians must understand both barriers to access and the unique needs of newcomer youth. As such, in this critical narrative review, the extant literature pertaining to school-based psychosocial support services for newcomer youth during this process of school integration was synthesized. This review was guided by a critical social justice lens and the following question, *what is currently known about the ways newcomer youth access and experience school-based psychosocial support services throughout the process of school integration?*

## Literature Review Process

### Critical Social Justice Lens

This literature review was guided by a critical social justice lens. Socially just research in the field of education and as it relates to children and youth calls for awareness of privilege in research and for an increasing consciousness around the ways in which our investigations can contribute to or mitigate existing inequities (Russell, 2016). Furthermore, research guided by this lens pursues justice and recognizes oppression (Stewart, 2014). According to Stewart (2014), social justice within educational and psychological research seeks to (1) investigate issues of inequity based on minoritized identities, (2) engage in critical inquiry across disciplines and with multiple perspectives, and (3) contribute to an overall goal of promoting the equal and full engagement of all groups of individuals. This lens represents a good fit for this critical narrative review, as newcomer youth are often marginalized and, because of their multiple intersecting identities, face social sidelining throughout the school integration process. These identities can include but are not limited to, English language proficiency, race, ethnicity, religious beliefs and practices, ability, gender, sexuality, and economic status. By incorporating a social justice perspective, this project is motivated by a desire to acknowledge and end oppression related to gender, race, ethnicity, sexual identity, ability, and socio-economic status (Stewart, 2014). This research intends to contribute to a growing body of work that is responsive to current needs and actively challenges dominant service models and structures to promote systemic change. In this review, this societal narrative was rejected; instead, there was an intent to synthesize and subsequently promote research that aligns with the social justice core values of agency, anti-racism, and equity.

### Critical Narrative Literature Review

To address the guiding question, a critical narrative literature review was conducted to determine what is currently known about the ways newcomer youth access and experience school-based psychosocial support services throughout the process of school integration. A narrative literature review was employed to collect and synthesize data to provoke thought and controversy on the topic, while being flexible in its approach (B. N. Green et al., 2006). Narrative reviews are broad in scope and methodology and are often critiqued for their unsystematic approach (B. N. Green et al., 2006). However, Byrne (2016) suggested that narrative reviews must follow specified criteria to ensure confidence in the conclusions drawn by the review. A narrative review can be defined as a summary, interpretation, and critique of current literature that seeks to derive theories and key concepts through analysis (Horsley, 2019). Narrative literature reviews also offer a critique and intend to provoke further thought on the subject of review (B. N. Green et al., 2006).

A critical narrative literature review is appropriate for this topic, as it provides flexibility for collecting, synthesizing, and representing the data on the subject matter (Gregory & Denniss, 2018). Specifically, it is appropriate for the topic of newcomer youths’ experiences of school-based psychosocial support services throughout the process of school integration, as it provides a broad overview of the existing research and allows for research of significance to be reviewed more comprehensively and to be included or excluded based on its relevance to the topic. This approach is flexible in nature, and formal procedures are non-existent in current literature. Gregory and Denniss (2018) provided guidelines for systematically conducting and writing a narrative review that were implemented at each stage of the review process. In response to these critiques, this review includes a description of its methodical approach to increase the rigor of the process and subsequent findings. Moreover, this review employs a critical social justice lens, and not only draws themes from extant research but also uses this lens to analyze the extant research, thereby employing it in its analysis of the current research. Therefore, a critical narrative review is best suited to determine what is currently known about newcomer youth access to school-based psychosocial support services, to offer a critique of the themes emerging from current evidence-based research, and to suggest ways forward for future research to examine this phenomenon.

### Inclusion and Exclusion Criteria

In line with the target population, this critical narrative review centered on research pertaining to the school integration of newcomer youth aged 15–24 and their access to school-based psychosocial support services. Articles and reports that were included cover a 20-year period, between 2002 and 2022, reflecting both recent trends in immigration and asylum seeking, as well as school integration and access to support services. Studies that focused on school integration in Canada were included. Some American and European studies were also included to provide a broad overview of current research and where findings were transferrable to newcomers in North America. Qualitative, quantitative, and mixed-methods research were included, as well as reports from the United Nations and the Canadian government; the review was limited to peer-reviewed scholarly publications in English. Research with adults over the age of 24 or children under 15 years was excluded. In line with the purpose of this review, studies that focused on other issues (e.g., immigration policy) and/or psychopathologies experienced by newcomer youth (e.g., clinical case studies) were excluded. Conference proceedings, book chapters, and editorials were also excluded.

### Literature Search Strategy

**Figure 1.**
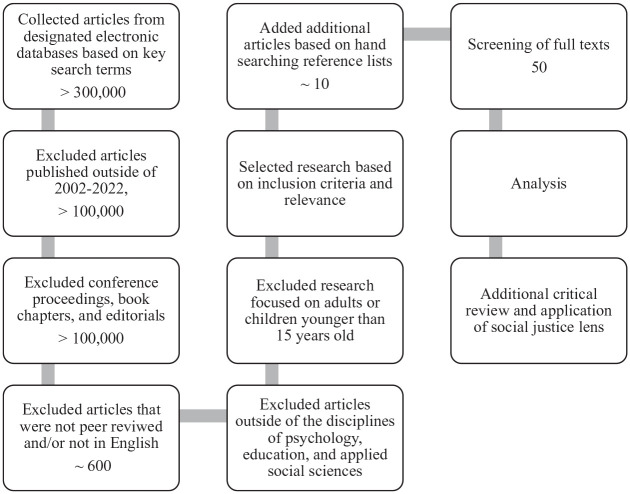
Flowchart of critical narrative literature review strategy.

An initial preliminary search was conducted to refine the search criteria and determine which studies would be included in the review. For the search, key terms such as *school integration, newcomer youth, immigrant youth, refugee students, international students, school-based support services, mental health services, and immigration* were used to locate research to be included in this narrative review. To capture early research in this area, *acculturation* was also added to search terms as an early descriptor of school integration. Several electronic databases, including PsycInfo, PubPsych, PubMed, Web of Science, SpringerOpen, JSTOR, Research Gate, and Google Scholar were used in conducting the search. These databases cover a broad area of research in psychology, educational psychology, education, and applied social sciences. Research studies were then individually evaluated based on the inclusion and exclusion criteria listed above and for their relevancy and significance to the topic of the review. Studies that employed a critical or social justice lens were included. Overall, 50 studies were included in this review based on their eligibility in meeting the inclusion criteria above and for their specificity in addressing access to support services and school integration for newcomer youth. Figure 1 illustrates the review process in more detail.

### Analysis Process

Studies that met the inclusion criteria underwent analysis. Each article or chapter was reviewed, with initial notes and themes developed. The list of initial descriptive themes was then synthesized to develop analytical themes that addressed the research question, captured the content of the studies more broadly, and aligned with the critical social justice lens (Thomas & Harden, 2008). In applying a critical social justice lens, priority was given to research that highlighted the voices of newcomer youth, was critical and/or social-justice-oriented in its approach, and did not perpetuate harmful stereotypes of newcomer youth. During analysis, themes were developed that align with these social justice values, including research that is interdisciplinary and promotes the full engagement of newcomer youth in society (Stewart, 2014).

### Current Research Findings on Access to School-Based Psychosocial Services for Newcomer Youth

Overall, the findings of this review allowed us to develop several themes which describe the experience of newcomer youth during the process of school integration. Moreover, these themes emerging from the literature directly impact newcomer uptake of school-based psychosocial support services and provide direction for future research. These themes include (a) underutilization and discrimination, (b) culture shift, (c) transition planning, and (d) lived experience. Table 1 provides an overview of the key themes that emerged from this review as well as key examples taken from recent research.

**Table 1.** Key Themes Across Extant Literature on Access to Support Services for Newcomer Youth During School Integration.

Theme	Examples
Underutilization and discrimination	Newcomers to Canada face social, cultural, religious, linguistic, geographic, economic, and systemic discrimination that contributes to their underuse of psychosocial support services (Thomson et al., 2015)
	Newcomer youth called for more resources and supports, and identified bullying, racism, insufficient orientation to new systems, language barriers and stress as barriers to accessing support services (A. C. Smith et al., 2022)
Culture shift	Newcomer youth were faced with navigating Westernized education systems and a fear of social rejection as barriers to successful school integration, including accessing English language instruction (J. Li, 2010)
	Support-service staff must place emphasis on the multiple, intersecting identities of newcomer youth in addition to providing culturally competent providers who acknowledge power differences, systems of oppression, and advocate for social action to support newcomer youth (Clarke & Wan, 2011)
Transition planning	Successful school integration has a direct impact on the pursuit of post-secondary education for newcomer youth and transition planning services, as well as academic support is needed for newcomer youth (Gallucci & Kassan, 2019)
	Newcomer youth also reportedly faced barriers to attaining career and post-secondary goals by spending increased time on work, English language learning, and academic responsibilities, leading to less opportunity to engage in other activities leading to career exploration and goal setting, including socializing with peers and engaging in extra curriculars (Ángeles, 2021)
Lived experience	Current research has identified general barriers to accessing support services but fails to capture the rich lived experiences of newcomer youth navigating both school integration and intervention services, particularly in school-based settings (Stermac et al., 2013)
	Research has indicated that an important aspect of social integration for newcomer youth was building peer relationships with other newcomers, as well as Canadian-born or long-term resident youth. Understanding the ways in which lived experience impacts these friendships, such as bonding over shared experiences as newcomers, is important for supporting the social adjustment of newcomer youth (Selimos et al., 2018)

### Underutilization and Discrimination

Underutilization and discrimination are consistently recognized as factors contributing to newcomer youths’ experiences of school-based psychosocial support services in the current literature. Forms of discrimination including social, cultural, religious, linguistic, geographic, economic, and systemic are acknowledged as limiting newcomer utilization of services and more broadly negatively impacting their experience of school integration (Thomson et al., 2015). Experiences of social and linguistic marginalization were identified by newcomer participants in an American study examining pathways to integration for underrepresented newcomer students (Oikonomidoy, 2015). Participants expressed frustration when trying to socialize and study alongside peers while not being able to speak English or Spanish, the languages of instruction in their school (Oikonomidoy, 2015). More specifically, newcomer youth from racialized backgrounds reported that they were often met with low expectations by teachers who may be stereotyping them, and this had a negative effect on their self-confidence and expectations for future opportunities (Selimos et al., 2018). Teachers and school communities more broadly were found in need of more training in intercultural competence and the unique needs of immigrant and refugee youth to meet their needs (Rossiter & Rossiter, 2009). Newcomer youth themselves noted that they were often the subject of cultural stereotyping, which can be a barrier to both accessing services and school integration broadly (Lee & Koro-Ljungberg, 2007; Matejko et al., 2021; Oikonomidoy, 2015).

In addition to discrimination occurring at multiple levels of the school integration process, underutilization is also a pervasive issue in the access and implementation of school-based psychosocial support services. A study investigating mental health outcomes for refugee youth found that barriers to access included a distrust of systems and authority, a pervasive stigma around accessing mental health services, the urgency of addressing resettlement stressors and a continued emphasis on cultural and linguistic barriers (Ellis et al., 2011). Gramaglia et al. (2016) also noted that language and cultural difference remain key barriers to accessing services and suggested the need for a linguistic or cultural mediator to assist in providing programming for newcomers. Selimos and Daniel (2017) interviewed newcomer youth who identified underutilized resources due to the lack of knowledge of availability and resources that they would like to see in their school communities. Supports identified by newcomer youth included peer mentorship activities (e.g., group work to meet youth who have been in Canada for longer periods), one-on-one support with educational assistants and other staff, anti-bullying services, and a greater variety of extra-curricular sports programming (Deckers & Zinga, 2010; Selimos & Daniel, 2017). In one study, newcomer youth were asked to share their experiences of their transition and provide advice for other newcomer youth, generating important recommendations for services. Newcomer youth called for more resources and supports and identified bullying, racism, insufficient orientation to new systems, language, and stress as barriers to accessing support services (A. C. Smith et al., 2022). Schools and school-based psychosocial support services were found not to fully meet the unique needs of newcomer students (A. C. Smith et al., 2022; Suárez-Orozco et al., 2010).

In addition to newcomer youth underutilizing services, service gaps were also identified in the literature. Early research in this area identified the importance of language programming for newcomer students, but existing school-based psychosocial services lacked specificity and cohesion with other programs already in place, so they were not as effective in supporting newcomer students (Short, 2002). A study conducted in Newfoundland and Labrador found that there were still service gaps for newcomer youth in the areas of transportation, academic bridging, transitional programs, and career counseling (X. Li et al., 2017). These two studies indicated that while newcomer youth themselves often articulate which support services are needed to assist them in their transition, underutilization and discrimination remain significant factors in deterring their use of school-based psychosocial support services. To effectively reach newcomer and refugee newcomer youth, S-BPSS must address barriers while focusing on both systematic outreach and screening (Brymer et al., 2008). Overall, research addressing themes of underutilization and discrimination regularly identifies stereotyping and discrimination faced by marginalized newcomer students and underutilization of existing services due to distrust, stigma, lack of specificity, and an inability to meet needs.

### Culture Shift

A significant theme in accessing support services for newcomer youth throughout the process of school integration is a shift in culture. The period of school integration and adjusting to a cultural shift often results in anxiety among newcomer students, which can impact their adjustment process and well-being (Wang & Mallinckrodt, 2006). Newcomer youth were faced with navigating Western education systems and a fear of social rejection as barriers to successful school integration, including accessing English language instruction (J. Li, 2010). Moreover, newcomer youth uniquely experience tension in navigating their own cultural identities as they are shaped by the Canadian context and for some newcomer youth, their desire to adopt a bi-cultural identity (Masinda et al., 2014; Matejko et al., 2021). In an arts-based engagement ethnographic study, Kassan et al. (2019) captured the experiences of one newcomer youth, who described several aspects of navigating a cultural shift throughout the process of school integration in Canada, including navigating social experiences at school and parental cultural values, which tended to conflict with those of her host country. Additionally, newcomer youth expressed difficulty adjusting to high school Canada and perceived an unwelcoming culture and climate in schools, in addition to stresses relating to parental expectations and cultural conflicts at home (Roderick et al., 2007). Difficulty understanding school systems across Canada, catching up, addressing gaps academically, and learning or improving English language skills were also reported by newcomer youth (Roderick et al., 2007).

Culture and the impact of family values was pervasive in the literature as an area in which newcomer youth struggle. Culturally diverse families and youth bring diverse methods of communication and values to their interactions with school staff, which can sometimes lead to cultural misunderstandings and conflict (Lee & Koro-Ljungberg, 2007). However, school programming was found to be more effective when parents are included in ways that are collaborative and culturally responsive. A mixed methods study of 12 newcomer youth found that participants wanted increased involvement and feedback from parents to support their school transition and a home that is more welcoming to friends to support their social transition (Burgos et al., 2019). These supports led to improved understanding, warmth, communication, and cultural integration at home, which supported newcomer youth in their overall transition in Canada (Burgos et al., 2019). Moreover, in a report by Khanlou et al. (2009), newcomer youth discussed the impact of family on their mental health and ability to access school-based supports, including parental employment, changes in family roles, family separations, and the role of family resilience. Changing cultural expectations and roles within the family, as well as adjusting to the Canadian cultural context, both played a role in the challenges identified by newcomer youth and thus informed their needs for mental health support services to address (Khanlou et al., 2009).

School-based psychosocial support services that are effective in supporting the school integration of newcomer youth are those that reach students and families in culturally responsive ways and that take into consideration beliefs, practices, and identities of those they serve (Bennouna et al., 2019). This approach represents a shift in programming from dictating acculturation and assimilation skills, such as language acquisition, to a more collaborative approach whereby programming can facilitate the learning of a host language through inclusion in a host school community (Allen, 2006). Mentorship programs and those addressing cultural guidance for families and newcomer youth were effective in supporting their overall inclusion and well-being (Gaytan et al., 2007). Clarke and Wan (2011) advocated for an anti-oppression approach when working with newcomer youth in schools in Canada and noted that support staff should bring multiple identities to their work with newcomer students, as well as recognize the agency and goal setting capacity of newcomer youth themselves (Clarke & Wan, 2011). Support-service staff must place emphasis on the multiple, intersecting identities of newcomer youth in addition to providing culturally competent providers who acknowledge power differences, systems of oppression, and advocate for social action to support newcomer youth (Clarke & Wan, 2011). Current educational research has advocated for a widening of the scope of multicultural education and culturally relevant pedagogy, to provide equitable education for newcomer youth and address these issues of navigating a cultural shift (Fruja Amthor & Roxas, 2016).

### Transition Planning

Research has solidified the link between school integration, transition planning, and the pursuit of post-secondary education and career opportunities after completing secondary school with universities as important sites of integration into Canadian society (Sinacore & Lerner, 2013). Successful school integration has a direct impact on the pursuit of post-secondary education for newcomer youth, and transition planning services and academic support are needed for newcomer youth (Gallucci & Kassan, 2019). Moreover, educational programs and experiences in secondary school must address skills training, career planning, job search skills, resume writing, employment mentoring, and English language deficits to effectively support newcomer youth (Gallucci & Kassan, 2019). Development and leadership programs for newcomer youth were also effective in supporting pathways to post-secondary goals by promoting critical thinking and decision-making skills (Shakya et al., 2010). Additional factors negatively impacting refugee newcomer youths’ pursuit of post-secondary education were found to be weak English or French language skills as well as poor grade placements and academic support (Wilkinson et al., 2012). Even when transition planning support services exist for newcomers, they do not consider the differences among minority groups and differing priorities for accessing post-secondary education and achieving educational success (Abada & Tenkorang, 2009).

Newcomer youth also reportedly faced barriers to attaining career and post-secondary goals by spending increased time on work, English language learning, and academic responsibilities. This led to less opportunity to engage in other activities related to career exploration and goal setting, including socializing with peers, and engaging in extracurriculars (Ángeles, 2021). A Canadian study utilized focus groups with 57 refugee newcomer youth to examine their career and educational goals after coming to Canada. Systemic barriers were pervasive in limiting newcomer youth from achieving their goals, pointing to a larger failure in the Canadian refugee settlement system (Shakya et al., 2012). This study highlighted systemic barriers and discrimination faced by refugee newcomer youth in attaining their academic goals and preparing for post-secondary careers and studies, including barriers to information, financial and linguistic barriers, and a limited recognition of schooling and credentials earned in their countries of origin (Shakya et al., 2012). Zhang (2016) found that international students found significant benefits to academic advising, including both academic and personal validation. However, they also experienced school counselors who had limited knowledge of international students and their experiences, as well as limited cultural competency, a lack of coordination with other services and professionals, and delayed course information, making their services unhelpful and invalidating for international students. The role of school staff, including teachers, mentors, and counselors remained an important source of support for newcomer students navigating these transitional plans when staff were culturally responsive and able to effectively meet the needs of newcomer students (Gallucci & Kassan, 2019).

According to Stewart (2014), school staff, such as counselors, are well placed to implement programming and support services that promote equity and social justice for newcomer students. Many newcomer students and their families arrive in Canada with an expectation and desire for increased opportunity, often in the form of education and post-secondary studies for their children. Providing resources and school-based psychosocial support services that support newcomer youth in navigating this increasingly complex system of college and university criteria, applications, and acceptance policies is another way of ensuring that newcomer youth have access to the same opportunities as their Canadian-born counterparts. Providing support services also aligns with a key priority in social justice research, which is promoting the equal engagement of all individuals in society (Stewart, 2014). There is also an increased need for career counselors at universities who are uniquely positioned to support newcomer students in their transition, to increase their knowledge of appropriate services, and increase their awareness of challenges faced by this student population (Sinacore & Lerner, 2013). Post-secondary staff must also increase their awareness of cultural, societal, and institutional barriers to academic success faced by newcomer youth and the ways in which these factors impact their academic success (Sinacore & Lerner, 2013). More needs to be done to support newcomer students’ success at both the secondary and post-secondary school levels. Overall, transition planning was highlighted as an area in which newcomer youth sought increased support from school-based psychosocial support services and an important factor in their school integration process and in providing opportunities for this group of students.

### Lived Experience

All themes impacting newcomers’ access to support services during the process of school integration are underscored by a pervasive need to capture, record, and learn directly from newcomer experiences. School-based settings are important for investigating and capturing the lived experiences of youth navigating intervention services (Crooks et al., 2021; Stermac et al., 2013). It is continually reaffirmed throughout the studies that the process of adaptation and integration for newcomers navigating a host culture is complex and focusing on individual and group lived experiences is key (Berry et al., 2006). Lived experiences highlight the ways in which newcomer youth experience their new communities and the ways in which the social and physical aspects of their new communities and schools contribute to their adjustment and integration (Van Ngo, 2009). The lived experiences of newcomer youth directly impact their experience both of school integration and school-based psychosocial support services. A mixed-methods study found that adverse family and school stressors interacted to pose a threat to the integration of newcomer youth and negatively impacted their adjustment (Patel et al., 2016). Moreover, professionals that work with newcomer youth can learn valuable insights from youth lived experiences and incorporate strategies into their practices to support school integration and settlement (Van Ngo, 2009). Newcomer youth are not a homogeneous group. They represent a vast number of experiences, cultures, languages, and needs. Acknowledging their unique and minoritized identities through lived experiences is a critical component of addressing issues of inequity within a critical social justice lens (Stewart, 2014). By understanding and acknowledging the unique lived experiences of newcomer youth, school-based psychosocial support services and the professionals that implement them can learn to be more responsive in their approaches.

An important aspect of social integration for newcomer youth was building peer relationships with other newcomers, as well as Canadian-born or long-term resident youth. Understanding the ways in which lived experience impacts friendships, such as bonding over shared experiences as newcomers, is important for supporting the social adjustment of newcomer youth (Selimos et al., 2018). Newcomer youth also reported difficulties developing relationships with Canadian-born or long-term resident youth, especially those in established peer groups. Furthermore, they cited linguistic insecurity, fear of being teased and experiences of racism, xenophobia, and Islamophobia as barriers to social interactions (Selimos et al., 2018). Studies that referenced the social aspect of school integration for newcomer youth noted that although newcomer youth require support, so too do their peers as both an untapped resource to support newcomer youth and as an intervention target to decrease racism and othering (Crooks et al., 2021; N. A. Smith et al., 2020). Crooks et al. (2021) examined school-based and peer-focused psychosocial support programming for newcomers and found that approaches involving teachers, peers, and newcomers themselves were effective in promoting an equity-based, whole school approach to social-emotional learning. Programs that included peer mentorship and teacher-led prejudice-reduction approaches were also effective in supporting the school integration of newcomers (Crooks et al., 2021).

Several studies examined the effects that school-based psychosocial support services had on newcomer youth and their lived experiences of the supports. Fazel et al. (2016) examined the experiences of refugee youth accessing school-based mental health services, with youth reporting anxiety and rumination around the asylum process (Fazel et al., 2016). School is a key setting for these services, as newcomer students felt safe in a familiar setting, and teachers played a key role in both referring and collaborating on these interventions (Fazel et al., 2016; Guruge & Butt, 2015). Pryce et al. (2019) examined the effects of an after-school group mentoring intervention for Canadian newcomer youth who were interviewed after participating in the club. The benefits of participating, including a sense of belonging, improved English language skills, and a connection with peers, were described (Pryce et al., 2019). Finally, an evaluation of the STRONG school-based group intervention in schools across Canada found that newcomer youth were increasingly engaged, connected, and developed resilience skills (Crooks et al., 2020). These findings reaffirm that newcomer youth who have access to effective services benefit from connection to peers and that their lived experiences are invaluable in developing and refining school-based psychosocial support services and program planning. Capturing the lived experiences of newcomer youth as they engage with school-based psychosocial support services is also crucial in program planning, design, and implementation and can support those working with newcomer youth in adapting their approaches to be both flexible and responsive to needs.

## Discussion

This critical narrative review contributes new understandings to what is currently known about the ways in which newcomer youth access and experience school-based psychosocial support services throughout the process of school integration. Four key themes that underscore the realities of newcomer youth in Canada: (a) underutilization/discrimination, (b) culture shift, (c) transition planning, and (d) lived experience were identified. While the themes are important in understanding the unique facets of the newcomer experience in accessing school-based psychosocial support services, readers should also be aware that they are interconnected, as several studies have affirmed the complexity and interconnectedness of each facet of school integration (Gaytan et al., 2007; Kassan et al., 2019; Lee & Koro-Ljungberg, 2007; Matejko et al., 2021). For example, studies across themes recognized the importance of peer and school staff relationships for supporting newcomer youth. Providing relationship-based supports emerged as a key factor, regardless of the nature of the support service. Taken together, the extant body of research reveals that newcomer youth face significant challenges throughout the process of school integration. Research investigating their experiences, as well as their impressions of and ability to access support services, is vital in supporting them through this process. Moreover, these themes highlight the necessity of capturing the lived experiences of newcomer youth, as their perspectives of discrimination and navigating a culture shift are individual and contain significant nuance.

In addition to providing an overview of extant literature, this review offers a critique through a social justice lens. Throughout the literature, newcomer youth (including international students and refugees) experience marginalization because of their cultural, linguistic, and religious diversity. Moreover, school staff and service providers who seek to implement school-based psychosocial support services for newcomer youth are lacking in cultural responsiveness and recognition of the diverse values, priorities, and communication styles that newcomers and their families bring to the school community. Although this research highlights these concerns, many studies fail to position newcomer youths’ experiences as central in moving toward an equitable approach to providing services. Many studies also do not situate themselves within a critical social justice lens for education, which inadvertently perpetuates existing colonial education systems and the systemic sidelining of newcomer youth within schools (Stewart, 2014).

This critical narrative literature review reflects what is currently available on the topic. To be comprehensive, a broad range of studies regarding newcomers’ experiences of school-based psychosocial support services were considered in this review. This review is also situated within a growing body of research that seeks to position newcomer youth as experts in their own experiences. In doing so, the research places value on the unique lived experiences of newcomer youth and the utility of their experiences in designing and implementing school-based psychosocial support services such as counseling, social groups, and English language interventions.

## Limitations and Directions for Future Research

This review is subject to certain limitations, including its process, criteria for study inclusion, and definition of terms. An effort was made to address common critiques of critical narrative literature reviews by ensuring its methodology and search process was clearly outlined. However, the nature of a narrative literature review is imperfect, and its purpose is not to synthesize every study available on the topic but rather to provide a synthesized summary of significant research and to offer a critique for future studies (B. N. Green et al., 2006). At the same time, supplementary systematic reviews or other methodological approaches may also contribute additional information to the overall understanding of current research on school-based psychosocial support services through the process of school integration.

This review is also limited in its inclusion and exclusion criteria. Several studies included in the review focused on specific geographical populations of newcomers or on newcomer access to a specific school-based psychosocial support service as opposed to broad findings on newcomer youth access to support services during school integration. Rather than limiting this review to a select number of studies, a comprehensive overview of extant literature was prioritized. Although these studies are beneficial to include, as they extend the themes drawn from the research, more research is needed that focuses on newcomer experiences more broadly in relation to accessing support services. Specifically, studies that examine newcomer youths’ experience of support services from their perspectives are needed to understand this complex phenomenon more fully. Additionally, several studies included in this review considered the experiences of newcomer youth, and several others reviewed interventions and school-based psychosocial supports, but very few incorporate the experiences of newcomer youth accessing and using these school-based psychosocial support services. This review was also limited in its definition of terms. For this review and to include as much of the extant research as possible, the definition of newcomer youth was expanded to include refugee youth and international students. Although both refugee youth and international students are considered to be newcomers, they also face a distinct set of challenges and have unique lived experiences, which are not captured separately in this review.

Future research must capture the lived experiences of newcomer youth accessing school-based psychosocial support services during the process of school integration. Although the current literature examines the challenges faced by newcomers and evaluates the school-based psychosocial support services available to them throughout school integration, it often fails to capture the experiences of newcomer youth themselves. Insufficient studies investigate the first-hand accounts of newcomer youth accessing support services while navigating school integration. Moreover, methodologies employed in existing research lack responsiveness and specificity to meet the needs of working with this group. Common approaches in the literature do not employ a social justice lens and subsequently do not consider the complex identities and inequities faced by newcomer youth. Moreover, these research approaches are typically colonial in origin and focus on newcomers as the subjects of research, rather than as collaborators and individuals with agency. Research focusing on newcomer youth must be socially just, reflexive, and actively prioritize the experiences of youth themselves. When newcomer youth can share their experiences directly, they are able to provide invaluable insight into this phenomenon.

### Relevance to School Psychology

School psychologists are challenged to be leaders in their communities and to advocate for the best interests of the students and families that they serve, including newcomer youth. As such, they need to advocate and provide culturally responsive and anti-oppressive services, and to support programming in schools that best meets the needs of newcomer youth (Clarke & Wan, 2011). School psychologists must support the school integration of newcomer youth and promote S-BPSS which are effective in meeting their needs. This includes both targeted and school-wide intervention programming to assist newcomer youth and promote their access to S-BPSS. School psychologists may also consider connecting newcomer youth and families to external resources, and/or advocating for those resources to be implemented in the school setting. Schools are often found to be the most accessible for newcomer youth and their families when the S-BPSS provided are culturally responsive and collaborative (Bennouna et al, 2019; Gaytan et al., 2007). These types of S-BPSS may include settlement services in schools, working with cultural brokers and translators, parent outreach workshops or EAL classes and orientation programs for new families to the school (Elizalde-Utnick, 2010; Suárez-Orozco & Marks, 2016). School psychologists can advocate for these types of services to make their schools more inclusive and their services more effective for newcomer youth and their families.

## Conclusion

The purpose of this review was to synthesize and offer a critical perspective on what is currently known about the ways newcomer youth access and experience school-based psychosocial support services throughout the process of school integration. Themes emerging from this research place an important emphasis on the lived experiences of newcomer youth, as well as their challenges and needs during school integration. Moreover, all the themes are deeply interconnected and reflect a growing need in the research to investigate not only the process of school integration and the implementation of school-based psychosocial support services but rather to develop a body of research based on the experiences of newcomer youth themselves. Research capturing the experiences of newcomer youth as they access services and navigate this transition is the next step in developing a comprehensive understanding of what is needed to better support newcomer youth in schools in Canada. This step includes implementing methodologies that are culturally responsive and socially just, as newcomers are often socio-politically marginalized. Newcomer youth continue to represent an important group in schools, and research capturing their experiences will strengthen the development of support services and place newcomer youth at the center of this work.

## References

[bibr1-08295735221130442] Abada T. Tenkorang E. Y. (2009). Pursuit of university education among the children of immigrants in Canada: The roles of parental human capital and social capital. Journal of Youth Studies, 12(2), 185–207. 10.1080/13676260802558870

[bibr2-08295735221130442] Allen D. (2006). Who’s in and who’s out? Language and the integration of new immigrant youth in Quebec. International Journal of Inclusive Education, 10(2–3), 251–263. 10.1080/13603110500256103

[bibr3-08295735221130442] Ángeles S. L. (2021). Exploring high school newcomer youths’ futures: Academic and career aspirations. Journal of Urban Learning, Teaching, and Research, 16(1), 3–22.

[bibr4-08295735221130442] Areepattamannil S. Freeman J. G. (2008). Academic achievement, academic self-concept, and academic motivation of immigrant adolescents in the greater Toronto area secondary schools. Journal of Advanced Academics, 19(4), 700–743.

[bibr5-08295735221130442] Bajaj M. Suresh S. (2018). The “warm embrace” of a newcomer school for immigrant & refugee youth. Theory into Practice, 57(2), 91–98. 10.1080/00405841.2018.1425815

[bibr6-08295735221130442] Bennouna C. Khauli N. Basir M. Allaf C. Wessells M. Stark L. (2019). School-based programs for supporting the mental health and psychosocial wellbeing of adolescent forced migrants in high-income countries: A scoping review. Social Science & Medicine, 239, 112558. 10.1016/j.socscimed.2019.112558 31539785

[bibr7-08295735221130442] Berry J. W. Phinney J. S. Sam D. L. Vedder P. (2006). Immigrant youth: Acculturation, identity, and adaptation. Applied Psychology, 55(3), 303–332. 10.1111/j.1464-0597.2006.00256.x

[bibr8-08295735221130442] Brymer M. J. Steinberg A. M. Sornborger J. Layne C. M. Pynoos R. S. (2008). Acute interventions for refugee children and families. Child and Adolescent Psychiatric Clinics of North America, 17(3), 625–640. 10.1016/j.chc.2008.02.007 18558316

[bibr9-08295735221130442] Byrne J. A. (2016). Improving the peer review of narrative literature reviews. Research Integrity and Peer Review, 1(1), 10–13. 10.1186/s41073-016-0019-2 29451529 PMC5803579

[bibr10-08295735221130442] Burgos M. Al-Adeimi M. Brown J. (2019). Needs of newcomer youth. Child and Adolescent Social Work Journal, 36(4), 429–437. 10.1007/s10560-018-0571-3

[bibr11-08295735221130442] Clarke J. Wan E. (2011). Transforming settlement work: From a traditional to a critical anti-oppression approach with newcomer youth in secondary schools. Critical Social Work, 12(1), 13–26.

[bibr12-08295735221130442] Crooks C. V. Hoover S. Smith A. C. G. (2020). Feasibility trial of the school-based STRONG intervention to promote resilience among newcomer youth. Psychology in the Schools, 57(12), 1815–1829. 10.1002/pits.22366

[bibr13-08295735221130442] Crooks C. V. Kubishyn N. Noyes A. Kayssi G. (2021). Engaging peers to promote well-being and inclusion of newcomer students: A call for equity-informed peer interventions. Psychology in the Schools. Advance online publication. 10.1002/pits.22623

[bibr14-08295735221130442] Deckers C. M. Zinga D. (2010). Locating home: Newcomer youths’ school and community engagement. Canadian Journal of Education, 35(3), 30–47.

[bibr15-08295735221130442] Elizalde-Utnick G. (2010). Immigrant families: Strategies for school support. Principal Leadership, 10(5), 12–16.

[bibr16-08295735221130442] Ellis B. H. Miller A. B. Baldwin H. Abdi S. (2011). New directions in refugee youth mental health services: Overcoming barriers to engagement. Journal of Child & Adolescent Trauma, 4(1), 69–85. 10.1080/19361521.2011.545047

[bibr17-08295735221130442] Fazel M. Garcia J. Stein A. (2016). The right location? Experiences of refugee adolescents seen by school-based mental health services. Clinical Child Psychology and Psychiatry, 21(3), 368–380. 10.1177/1359104516631606 26907460

[bibr18-08295735221130442] Fruja Amthor R. Roxas K . (2016). Multicultural education and newcomer youth: Re-imagining a more inclusive vision for immigrant and refugee students. Educational Studies, 52(2), 155–176. 10.1080/00131946.2016.1142992

[bibr19-08295735221130442] Gallucci A. Kassan A. (2019). “Now what?”: Exploring newcomer youth’s transition from high school to postsecondary education. Canadian Journal of Counselling and Psychotherapy, 53(1), 39–58.

[bibr20-08295735221130442] Gaytan F. X. Carhill A. Suarez-Orozco C. (2007). Understanding and responding to the needs of newcomer immigrant youth and families. The Prevention Researcher, 14(4), 10.

[bibr21-08295735221130442] Gramaglia C. Gambaro E. Rossi A. Toso A. Feggi A. Cattaneo C. I. Castignoli G. Mainini P. Tarricone I. Torre E. Zeppegno P. (2016). Immigrants’ pathways to outpatient mental health: Are there differences with the native population? Journal of Immigrant and Minority Health, 18(4), 878–885. 10.1007/s10903-015-0336-4 26705107

[bibr22-08295735221130442] Green B. N. Johnson C. D. Adams A. (2006). Writing narrative literature reviews for peer-reviewed journals: Secrets of the trade. Journal of Chiropractic Medicine, 5(3), 101–117. 10.1016/S0899-3467(07)60142-6 19674681 PMC2647067

[bibr23-08295735221130442] Gregory A. T. Denniss A. R. (2018). An introduction to writing narrative and systematic reviews—Tasks, tips and traps for aspiring authors. Heart, Lung & Circulation, 27(7), 893–898. 10.1016/j.hlc.2018.03.027 29857977

[bibr24-08295735221130442] Guruge S. Butt H. (2015). A scoping review of mental health issues and concerns among immigrant and refugee youth in Canada: Looking back, moving forward. Canadian Journal of Public Health, 106(2), e72–e78. 10.17269/CJPH.106.4588 PMC6972226 25955675

[bibr25-08295735221130442] Horsley T. (2019). Tips for improving the writing and reporting quality of systematic, scoping, and narrative reviews. The Journal of Continuing Education in the Health Professions, 39(1), 54–57. 10.1097/CEH.0000000000000241 30789378

[bibr26-08295735221130442] Immigrants, Refugees, and Citizenship Canada. (2018). 2018 Annual report to parliament on immigration. Ottawa: Government of Canada. https://www.canada.ca/content/dam/ircc/migration/ircc/english/pdf/pub/annual-report-2018.pdf

[bibr27-08295735221130442] Immigrants, Refugees, and Citizenship Canada. (2020a). 2019 Annual report to Parliament on immigration. Government of Canada. https://www.canada.ca/content/dam/ircc/migration/ircc/english/pdf/pub/annual-report-2020-en.pdf

[bibr28-08295735221130442] Immigrants, Refugees, and Citizenship Canada. (2020b). Supplementary information for the 2021–2023 Immigration Levels Plan. Government of Canada. https://www.canada.ca/en/immigration-refugees-citizenship/news/notices/supplementary-immigration-levels-2021-2023.html

[bibr29-08295735221130442] Kassan A. Priolo A. Goopy S. Arthur N. (2019, October 26–28). Investigating migration through the phenomenon of school integration: Anaya’s experience of resettlement in Canada [Conference Session]. Canadian Counselling Psychology Conference, Calgary, AB, (pp. 41–55).

[bibr30-08295735221130442] Khanlou N. Shakya Y. Muntaner C. (2009). Mental health services for newcomer youth: Exploring needs and enhancing access. The Provincial Centre of Excellence for Child and Youth Mental Health. https://accessalliance.ca/wp-content/uploads/2018/06/FINAL-REPORT-RG-No-122-CHEO-July-31-2009.doc.pdf

[bibr31-08295735221130442] Lee I. Koro-Ljungberg M. (2007). A phenomenological study of Korean students’ acculturation in middle schools in the USA. Journal of Research in International Education, 6(1), 95–117.

[bibr32-08295735221130442] Li J. (2010). ‘My home and my school’: Examining immigrant adolescent narratives from the critical sociocultural perspective. Race, Ethnicity and Education, 13(1), 119–137. 10.1080/13613320903550154

[bibr33-08295735221130442] Li X. Que H. Power K. (2017). Welcome to “the Rock”: Service providers’ views on newcomer youth integration in Newfoundland and Labrador. Journal of International Migration and Integration, 18(4), 1105–1122. 10.1007/s12134-017-0520-6

[bibr34-08295735221130442] Masinda M. T. Jacquet M. Moore D. (2014). An integrated framework for immigrant children and youth’s school integration: A focus on African Francophone students in British Columbia – Canada. International Journal of Education, 6(1), 90. 10.5296/ije.v6i1.4321

[bibr35-08295735221130442] Matejko E. Saunders J. F. Kassan A. Zak M. Smith D. Mukred R. (2021). “You can do so much better than what they expect”: An arts-based engagement ethnography on school integration with newcomer youth. Journal of Adolescent Research. Advance online publication. 10.1177/07435584211056065 PMC11055683 38686118

[bibr36-08295735221130442] Oikonomidoy E. (2015). Being the only one: Integration experiences of underrepresented newcomer students. Journal of Language, Identity, and Education, 14(5), 319–335. 10.1080/15348458.2015.1090779

[bibr37-08295735221130442] Patel S. G. Clarke A. V. Eltareb F. Macciomei E. E. Wickham R. E. (2016). Newcomer immigrant adolescents: A mixed-methods examination of family stressors and school outcomes. School Psychology Quarterly, 31(2), 163–180. 10.1037/spq0000140 27243242

[bibr38-08295735221130442] Pryce J. M. Kelly M. S. Lawinger M. (2019). Conversation club: A group mentoring model for immigrant youth. Youth & Society, 51(7), 879–899. 10.1177/0044118X18780526

[bibr39-08295735221130442] Roderick K. Janzen R. Ochocka J. Jenkins J. (2007). Pathways to success in Waterloo region: Immigrant youth at high school. Our Diverse Cities, 4(1), 139–144.

[bibr40-08295735221130442] Rossiter M. J. Rossiter K. R. (2009). Diamonds in the rough: Bridging gaps in supports for at-risk immigrant and refugee youth. Journal of International Migration and Integration, 10(4), 409–429. 10.1007/s12134-009-0110-3

[bibr41-08295735221130442] Rousseau C. Drapeau A. Lacroix L. Bagilishya D. Heusch N. (2005). Evaluation of a classroom program of creative expression workshops for refugee and immigrant children. Journal of Child Psychology and Psychiatry, 46(2), 180–185. 10.1111/j.1469-7610.2004.00344.x 15679526

[bibr42-08295735221130442] Russell S. T. (2016). Social justice, research, and adolescence. Journal of Research on Adolescence, 26(1), 4–15. 10.1111/jora.12249 27307689 PMC4905581

[bibr43-08295735221130442] Selimos E. D. Daniel Y. (2017). The role of schools in shaping the settlement experiences of newcomer immigrant and refugee youth. International Journal of Child, Youth & Family Studies, 8(2), 90. 10.18357/ijcyfs82201717878

[bibr44-08295735221130442] Selimos E. D. George G. (2018). Welcoming initiatives and the social inclusion of newcomer youth: The case of Windsor, Ontario. Canadian Ethnic Studies, 50(3), 69–89. 10.1353/ces.2018.0023

[bibr45-08295735221130442] Shakya Y. B. Guruge S. Hynie M. Akbari A. Malik M. Htoo S. Khogali A. Mona S. A. Murtaza R. Alley S. (2012). Aspirations for higher education among newcomer refugee youth in Toronto: Expectations, challenges, and strategies. Refuge: Canada’s Journal on Refugees, 27(2), 65–78.

[bibr46-08295735221130442] Short D. J. (2002). Newcomer programs: An educational alternative for secondary immigrant students. Education and Urban Society, 34(2), 173–198.

[bibr47-08295735221130442] Sinacore A. L. Lerner S. (2013). The cultural and educational transitioning of first-generation immigrant undergraduate students in Quebec, Canada. International Journal for Educational and Vocational Guidance, 13(1), 67–85. 10.1007/s10775-013-9238-y

[bibr48-08295735221130442] Smith A. C. Crooks C. V. Baker L. (2022). “You have to be resilient”: A qualitative study exploring advice newcomer youth have for other newcomer youth. Child and Adolescent Social Work Journal. Advance online publication. 10.1007/s10560-021-00807-3

[bibr49-08295735221130442] Smith N. A. Brown J. L. Tran T. Suárez-Orozco C. (2020). Parents, friends and immigrant youths’ academic engagement: A mediation analysis. International Journal of Psychology, 55(5), 743–753. 10.1002/ijop.12672 32285451

[bibr50-08295735221130442] Statistics Canada. (2017a). Census in Brief: Children with an immigrant background: Bridging cultures. Author. https://www12.statcan.gc.ca/census-recensement/2016/as-sa/98-200-x/2016015/98-200-x2016015-eng.cfm

[bibr51-08295735221130442] Stermac L. Clarke A. K. Brown L. (2013). Pathways to resilience: The role of education in war-zone immigrant and refugee student success (pp. 211–220). Springer New York. 10.1007/978-1-4614-6375-7_15

[bibr52-08295735221130442] Stewart J. (2014). The school counsellor’s role in promoting social justice for refugee and immigrant children. Canadian Journal of Counselling and Psychotherapy, 48(3), 251–269.

[bibr53-08295735221130442] Suárez-Orozco C. Gaytán F. X. Bang H. J. Pakes J. O’Connor E. Rhodes J. (2010). Academic trajectories of newcomer immigrant youth. Developmental Psychology, 46(3), 602–618. 10.1037/a0018201 20438174

[bibr54-08295735221130442] Suárez-Orozco C. Marks A. K. (2016). Immigrant students in the United States: Addressing their possibilities and challenges. In Banks J. A. Su.rez-Orozco M. M. Ben-Pertez M. (Eds.), Global migration, diversity, and civic education: Improving policy and practice (pp. 107–131). Teachers College Press.

[bibr55-08295735221130442] Thomas J. Harden A. (2008). Methods for the thematic synthesis of qualitative research in systematic reviews. BMC Medical Research Methodology, 8(1), 45–45. 10.1186/1471-2288-8-45 18616818 PMC2478656

[bibr56-08295735221130442] Thomson M. S. Chaze F. George U. Guruge S. (2015). Improving immigrant populations’ access to mental health services in Canada: A review of barriers and recommendations. Journal of Immigrant and Minority Health, 17(6), 1895–1905. 10.1007/s10903-015-0175-3 25742880

[bibr57-08295735221130442] United Nations Department of Economic and Social Affairs. (2008). Definition of youth [Fact sheet]. Author. https://www.un.org/esa/socdev/documents/youth/fact-sheets/youth-definition.pdf

[bibr58-08295735221130442] Van Ngo H . (2009). Patchwork, sidelining and marginalization: Services for immigrant youth. Journal of Immigrant & Refugee Studies, 7(1), 82–100. 10.1080/15562940802687280

[bibr59-08295735221130442] Wang C. D. Mallinckrodt B. (2006). Acculturation, attachment, and psychosocial adjustment of Chinese/Taiwanese international students. Journal of Counseling Psychology, 53(4), 422–433. 10.1037/0022-0167.53.4.422

[bibr60-08295735221130442] Wilkinson L. Yan M. C. Tsang A. K. T. Sin R. Lauer S. (2012). The school-to-work transitions of newcomer youth in Canada. Canadian Ethnic Studies, 44(3), 29–44.

[bibr61-08295735221130442] Zhang Y. (2016). An overlooked population in community college: International students’ (in)validation experiences with academic advising. Community College Review, 44(2), 153–170. 10.1177/0091552116633293

